# Community based service providers' perspectives on frequent and/or avoidable admission of older people with chronic disease in rural NSW: a qualitative study

**DOI:** 10.1186/1472-6963-11-265

**Published:** 2011-10-12

**Authors:** Jo M Longman, Judy B Singer, Yu Gao, Lesley M Barclay, Megan E Passey, Julie P Pirotta, Kathy E Heathcote, Dan P Ewald, Vahid Saberi, Paul Corben, Geoffrey G Morgan

**Affiliations:** 1 University Centre for Rural Health, PO Box 3074, Lismore, NSW 2480, Australia; 2 Northern Rivers General Practice Network, PO box 519 Lismore, NSW 2480, Australia; 3 North Coast Area Health Service, Locked Mail Bag 11, Lismore, NSW 2480, Australia

## Abstract

**Background:**

Frequent and potentially avoidable hospital admission amongst older patients with ambulatory care sensitive (ACS) chronic conditions is a major topic for research internationally, driven by the imperative to understand and therefore reduce hospital admissions. Research to date has mostly focused on analysis of routine data using ACS as a proxy for 'potentially avoidable'. There has been less research on the antecedents of frequent and/or avoidable admission from the perspectives of patients or those offering community based care and support for these patients. This study aimed to explore community based service providers' perspectives on the factors contributing to admission among older patients with chronic disease and a history of frequent and potentially avoidable admission.

**Methods:**

15 semi-structured interviews with community based providers of health care and other services, and an emergency department physician were conducted. Summary documents were produced and thematic analysis undertaken.

**Results:**

A range of complex barriers which limit or inhibit access to services were reported. We classified these as external and internal barriers. Important external barriers included: complexity of provision of services, patients' limited awareness of different services and their inexperience in accessing services, patients needing a higher level or longer length of service than they currently have access to, or an actual lack of available services, patient poverty, rurality, and transport. Important internal barriers included: fear (of change for example), a 'stoic' attitude to life, and for some, the difficulty of accepting their changed health status.

**Conclusions:**

The factors underlying frequent and/or potentially avoidable admission are numerous and complex. Identifying strategies to improve services or interventions for this group requires understanding patient, carer and service providers' perspectives. Improving accessibility of services is also complex, and includes consideration of patients' social, emotional and psychological ability and willingness to use services as well as those services being available and easily accessed.

## Background

The continued increase in hospital admissions is a significant and complex issue facing health services internationally, nationally and in local jurisdictions such as NSW. Reducing admissions that may be avoidable by exploring admissions of patients with ambulatory care sensitive conditions (ACS) has consequently been a major topic of research since the late 1980s [1,2]. Ambulatory care-sensitive (ACS) conditions are those "...*considered to be responsive to prophylactic or therapeutic interventions deliverable in the primary care setting, i.e. conditions that, with appropriate primary care, should not become serious enough to require admission to a hospital." *[3] p.1. Older patients with chronic conditions make the greatest contribution to ACS admissions [3-6].

In Australia, the rate of hospitalisation for ACS conditions is particularly burdensome in rural and remote areas. For example, in 2006-7 the age and sex adjusted rate of potentially preventable hospitalisations for chronic conditions (excluding diabetes) per 1,000 population was 8.1 in major cities compared to 15.6 in very remote regions [7].

There is some evidence that primary care interventions targeting patients with specific ACS chronic conditions can result in reductions in admissions [8-10], particularly for COPD and asthma [11]. This suggests that some admissions may indeed be avoidable, although few studies have used hospital admission as an outcome measure [11], and generally *"...the current evidence is too sparse to make general assertions that any form of intervention or treatment constitutes 'best practice'." *[6] p.3

There remains much uncertainty about how to define an avoidable admission and what the contributing factors are, particularly in rural settings. To date, research on avoidable admission has predominantly been quantitative, focussed on analyses of aggregated routine data sources using ACS conditions as a proxy for "avoidability". Notably, and as highlighted by Resar et al [12], this work has been reported from the perspective of providers of acute care services. There has been less research on frequent and/or avoidable admission beyond the broad ACS definition, especially from the perspectives of patients or those offering community based care and support for these patients [6,13]. These perspectives will provide a more comprehensive understanding of the antecedents to admission, and what might be needed to improve patient care, and whether rural settings pose particular challenges for patients and their primary health care providers.

This paper reports the findings of a qualitative study of the perspectives of rural community based providers and an Emergency Department (ED) physician, which coalesce around access to community based services. The study is part of a larger project by the authors exploring factors relating to frequent and/or avoidable admission amongst older patients with chronic disease. The authors are health service managers, clinicians and rural health researchers. This paper describes and explains the important internal barriers (as well as external barriers) to accessing services by these patients which are poorly described elsewhere yet are highly relevant to the demand side of the supply/need/demand landscape of rural community based services.

### Study site

This study took place in northern NSW, Australia; the geographic setting for the project as a whole. The area covers about 35,570 square kilometres on a 500 kilometre-long coastal strip along the NSW north coast, sharing a state border with Queensland. The population is around half a million which is mostly concentrated in regional cities and towns along the coastal fringe with the remaining population scattered across the rural hinterland, with associated difficulties in delivering and accessing health services. The area has a relatively large indigenous population (3.9% compared to State average of 2.1%), low socioeconomic status across a range of measures, a high proportion of people aged 65 years and over (18% compared to State average of 14%) and a rapidly increasing population of older people [14].

We interviewed professionals providing community based services for this patient group and an ED physician, from a small city, a small town and a more rural area to simulate the range of settings likely to be found in rural areas throughout Australia.

## Methods

We used semi-structured interviews addressing:

• the individual, social, environmental, economic and health service utilisation characteristics of older people with chronic conditions who have a history of frequent hospital admissions

• the factors which contribute to these patients being frequently admitted or having an admission which might be deemed to be avoidable

• what interventions or services currently exist that are effective in keeping these types of patients out of hospital

• whether there are any gaps in availability of primary care services or non-clinical care or type of care provided, or coordination of care provided, which contribute to admission.

In answering our questions we asked interviewees to specifically focus on certain types of patients: those over 65, with at least one chronic condition, who were non-Indigenous, not in palliative care and who had a history of frequent hospital admissions i.e. had at least three unplanned acute admissions in a 12 month period. This defined the cohort of patients in our study population for the wider project.

Semi-structured interviews were chosen as they can provide a supportive, flexible and comfortable process through which to elicit narrative description of experience and allow participants to reflect on and express opinion.

### Recruitment

Participants were recruited using criterion based purposive sampling [15] where participants were invited based on their geographic location, organisation and job role. Systematic selection of appropriate organisations and job role was initially guided by the literature on frequent and/or avoidable admission and expertise of local health service managers, clinicians and researchers on the project steering committee. Our aim was to ensure a broad range of experience and opinion was included in the study, to enhance our understanding of characteristics and factors contributing to hospitalisation amongst this patient group as described by professionals working with these patients on a daily basis.

Working through existing networks of colleagues, 16 individuals were approached by email and then a follow-up telephone call was made. Only one potential participant declined to be interviewed as they did not consider the time investment required was appropriate given their other work commitments. In this way we ensured that we included one experienced ED physician, and participants from a range of community based organisations, roles and geographic locations that represented key services to this group of patients. We also added new participants to our list of targeted participants based on suggestions from early interviewees.

In some cases we were fortunate to recruit individuals who were able to speak from a position of knowledge and experience of more than one organisation and role. Recruitment ceased when we reached saturation, i.e. when we stopped gathering new information, experience or opinions through subsequent interviews. We interviewed 2 men and 13 women. Table 1 gives a breakdown of work roles and organisations.

**Table 1:** Work role and organisation characteristics of participants

Main Category Current post	
Community nursing	

	• Community nurse
	• Pulmonary rehabilitation nurse in the community Heart failure nurse in the community

Physiotherapy	

	• Community based physiotherapist
	• Physiotherapist in a day therapy unit

Occupational Therapy	

	• Manager of home modifications assessment, implementation and management
	• Community occupational therapist

Admitting doctors	

	• ED admitting physician
	• GP/Visiting Medical Officer

Pharmacy	

	• Community pharmacist (conducting home medicines reviews as well as working in a community based pharmacy)

NGOs	

	• Meals on Wheels manager
	• Community transport managers

Home-based aged care provision	

	• Case manager for assessment, implementation and management of individuals' aged care provision in the home
	• Manager for assessment, implementation and management of individuals' aged care provision in the home
	• Transition care manager (from hospital to home)

### Development of the interview schedule

The interview topic areas were based on the literature on frequent admission and avoidable admission (not necessarily with a comparable group of patients) and our key research questions for this study. Interviews followed a semi-structured format which allowed for some flexibility in asking supplementary questions for clarification and further detail/examples. Interviews commonly lasted around 45 minutes.

### Data Collection

Interviews were conducted during 2010 at the University Centre for Rural Health in Lismore, NSW, or at the interviewee's place of work, and in one case, at the interviewee's home. Interviews were all digitally recorded with consent from the participants and recordings stored securely. The study was granted ethical approval from the North Coast Area Health Service Human Research Ethics Committee.

### Data analysis

NVivo 8 [15], was used to manage the project, manage the data, and to facilitate the transcription, coding and thematic analysis of data. Data analysis took place throughout the data gathering phase in an iterative and team-oriented process.

Initially, relevant literature was investigated and a broad set of categories defined. These were: age, carer, gender, health status, medication, mental health, rurality, self-management, social isolation, access to services, and socio-economic status. The research team then conducted interviews, and further categories were added as a result of interview data, including: falls, maintenance (the need for programs of support to be ongoing rather than short-term) and transport (availability and accessibility).

After careful listening, a summary document was produced of each interview using selective verbatim transcription combined with an initial interpretive review drawing out material which fitted identified categories and material which required amended and additional categories. Braun and Clarke [16] describe this approach as *"'theoretical' thematic analysis" *p. 84. It is an approach which reflects the analytical interest in the topic and focuses on a specific level of meaning, in this case the explicit meaning in participants' responses. It is considered appropriate for exploratory and descriptive studies.

The interpretive work was then consolidated with input from another team member and discussed at many team meetings. The categories eventually became the coding scheme. The summary document was then coded in NVivo 8 and further adjustments to the coding scheme made, enabling a much more detailed and nuanced categorisation, and articulation of the main themes and links across themes.

## Results

Material in quote marks and italic type represents verbatim quotation from the participants, rather than our interpretation.

### General observations about and from participants

Our participants were keen to participate in the interviews and to offer their opinions, knowledge and experience on this subject. For many, it was apparent by their expressed emotion and the stories they chose to tell that they were deeply affected by their work.

Participants universally agreed that this is a complex client group. The client group present with unique circumstances while also dealing with numerous and confronting challenges of the later stages of ageing, including their often rapidly declining health and loss of independence.

Although participants identified an intricate set of interrelated factors which influence frequent and potentially avoidable admission of this patient group, this paper will focus on one overarching theme which emerged; that is access to services. This theme linked other sub-elements of the analysis together. Services are defined here as community based, both clinical such as community nursing or physiotherapy, and non-clinical such as meals on wheels.

### Community based services for older patients with chronic disease

Reports of community based services available for this group of patients revealed the intricacies of service provision (multiple programs and providers, variation in eligibility criteria etc.), and associated difficulties of communication and coordination both within and between services. There was a good deal of misinformation in circulation about services and programs. For example, participants reported that often patients remain unaware of the different services and what they offer, and that commonly there are many different health and home care staff involved with one patient.

It was also frequently reported to us however, that in many cases *accessing *existing services is highly problematic and is influenced by a multiplicity of factors. One of these centres on the availability of services.

### Service availability

All of our participants described circumstances where services were not available due to lack of capacity, or lack of flexibility to deliver services when, where or how much patients need them.

*"If we want to keep people out of hospital we don't always have the capacity to do so. The main issue is access. The services are there, but they can't offer enough of their service, it's very limited"*. [Occupational Therapy]

Some explained that even initial, limited access to services can be problematic; and that this patient group often need a higher level or greater amount of service than they currently have access to.

*"For example, OT [occupational therapy] services are one day per month. People were on the waiting list for over 4 months for an OT assessment. So by the time they get to see the practitioner, their original problem is now a very serious condition." *[Occupational Therapy]

Lack of available community based services (either clinical such as occupational therapy or physiotherapy, or non-clinical such as homecare and meals on wheels), have an effect on patients physical and mental health state. They also contribute substantially to patients' "support systems" as referenced in the quotation below, and therefore potentially impact on frequent and/or potentially avoidable admission.

*"The biggest thing about patients staying out of hospital who've got chronic illness is if they've got really good support systems they tend not to readmit." *[Community Nursing]

A number of home based clinical and non-clinical services are only accessible to patients once they have had a hospital admission, which is possibly unfortunate given their potential to impact on admission.

### Service delivery models and the need for maintenance

Participants identified services such as community nurse led cardiac rehabilitation programs as effective in reducing admissions in this patient group as they offered intensive and consistent support over a number of weeks. However, they also identified difficulties in the finite timeframe of a few weeks of contact per patient offered by these programs. Many patients require long-term regular on-going support to benefit from these programs. This can be either in terms of sustaining their motivation and capacity for self-management, and/or to account for the fact that these patients often have poor short-term memory and some dementia.

Although availability of services is a critical aspect of accessing services, one important observation made repeatedly by our participants was that access is also determined by patients' ability, and willingness, to engage with available services and programs. This is discussed below.

### Service accessibility

#### Poverty and access to services

The issue of poor financial status was a common theme through most of the interviews and was linked to physical and mental health status and risk of admission. One participant in particular spoke evocatively about this concern. She described patients with few financial resources as 'stoic' and 'battling through' their lives, by problem-solving.

*"You find people turning off their power because they get really big electricity bills ... They talk about their pension not covering their basic needs ... they are not bathing because they don't want to use the hot water ... a lot of these people don't do well with heat, so they are all panicking now because they have little air conditioners and they are saying they won't be able to use them and you'll find there'll be a lot of people who'll go back to hospital just to get cool" *[Home based Aged Care provider]

This was also reflected by a community pharmacist who talked about this generation having a strong sense of pride and being "battlers".

The point was also made that chronic illness is associated with continued and rising expense and an increased need for additional services:

*"Once you are chronically ill there tends to be a lot of on-going expenses" *[Community Nursing]

Unsurprisingly, lack of finance was reported to be a factor in frequent admission in terms of restricting access to existing community based services, and to medication. Our participants discussed how individual financial circumstances impact on choices older patients make about the services they are able and willing to invest in which might support them and in turn reduce their risk of frequent and/or avoidable admission:

*"If they have to pay for home care then they don't have home care... and they end up in hospital more often...if they can't afford to see their doctor, or they can't get into their doctor, they end up in emergency." *[Community Nursing]

Lack of finance also influenced patients' ability to pay for transport to services considered important to their health needs, including transport to the GP and to the pharmacy to get prescriptions filled. This is exacerbated for those patients who lack the assistance of a carer or family member to go to the pharmacy on their behalf. Some patients were reported as being unable to afford the community bus.

Some participants identified reluctance amongst this patient group to buy in a homecare services package or similar (even with Government subsidy) and described patients trying to "*struggle on with the neighbour helping out"*. Our respondents reported that there is no support at home for these patients, and when an acute episode then occurs it becomes more difficult to discharge them from hospital in the absence of a robust support infrastructure, either creating a longer length of stay in hospital or "revolving door" patients particularly when access to assessment for services is also limited.

*"It's like a revolving door they just come into hospital and out of hospital and it goes around and around, it's three, or four times before they get assessed for nursing home"*. [Community Nursing]

#### Rurality

One other factor affecting patients' ability to access services relates to rural nature of the North Coast of NSW region in which the study was located, and the concomitant geographical isolation, relatively small population sizes, and disparate and limited availability of services, both clinical and non-clinical. Around two thirds of our participants discussed this issue specifically.

*"I really do feel that people in rural areas are so disadvantaged. There are very minimal services that go out to them and then to access the services that are available there are always transport and time issues and they will impact on the person's physical status as well as their psychological status"*. [Occupational Therapy]

This was balanced by some of our participants reporting that patients living in a place with a strong sense of community might have less perceived need for formal services such as homecare. They explained that in more rural areas people feel they are (or actually are) part of an extended family where there is less reserve in stepping in to help out others in need. In this scenario, a neighbour or a family member is more likely to drop in for a chat or to bring a meal.

Many of our participants also discussed the limited access to transport as an issue particularly affecting rural areas and impacting on access to services. Patients without a person to drive them were frequently reported as being unable to access services. Also the type of transport which might be available can be problematic. For example, a patient might be able to tolerate a two hour journey if they are sitting in a comfortable front seat, but if they are in a small uncomfortable bus this can be a real problem and worsen their pain. Transport issues therefore become more significant in rural areas where difficulties are exacerbated by greater distances to reach services.

#### Inexperience of services and how to access them

Some of our participants drew attention to patients' ability to access existing services in terms of understanding who and how to ask, and this was related to discussion about the complexity of services and providers. An unwell, older patient with poor support infrastructure at home will inevitably struggle to make sense of and access services they may be eligible for. This situation is exacerbated by poor memory for appointments, any dementia and the unorganised and chaotic lives some of these patients live. One participant stressed the problems in establishing services for these patients focusing on patients' lack of experience:

*"...but they've never accessed services before and they don't know how to go about it. So a big issue is getting services in place for people and often they just don't have the knowledge and they don't know what's out there and how to organise themselves"*. [Home based Aged Care provider]

#### Reluctance to access services

One important observation made by our participants was that accessibility is also determined by patients' emotional and psychosocial ability and willingness to utilise services which are available. An unexpected finding from this study was what we have termed 'reluctance' in patients. Patients were reported to be reluctant in three different but closely related ways described below.

#### Not ready to accept changes/services

In some situations, participants reported a deep-seated reluctance amongst this patient group to access services including home modification, home help or meals on wheels even when these services were available and accessible. In some cases this was reported to reflect a state of denial about 'not coping' with activities of daily living, and a reluctance to accept their changed health status.

*"I think one of the really difficult things is about acceptance. Acceptance of the disease process." *[Occupational Therapy]

Our participants reported that for some of these patients, accepting home modifications or help with day to day chores can represent a deeply confronting move, forcing them to acknowledge their changing mental or physical health status and ultimately their own mortality.

For many older people change is difficult to deal with and the fear of change can be paramount in their decision making processes, partly because change often relates to health deteriorating and an associated loss of independence. One participant put it like this - that change can be:

*"... so much more difficult to cope with... because change relates to health deteriorating... each change is about... loss of the person that they were. These become very powerful issues for the elderly." *[Occupational Therapy]

#### Maintenance of independence

Some of our participants identified a barrier to accessing services as stemming from an internal distress related to losing independence.

One participant described the link between accepting the Meals on Wheels service and loss of independence.

*"They want to be independent. The first sign of losing your independence is coming onto Meals on Wheels. It is quite an emotional thing; it is not such a straight forward thing as people think. They can't cook anymore. I can't go out in the car and get things anymore. I've lost my licence now and lost my independence." *[NGO]

A number of participants identified a generational issue of stoicism which for many stems from living through the depression and having learnt survival coping strategies. Maintenance of independence and control over their lives is therefore an important intrinsic part of their identity and mental health status.

*"It is generational, it is humiliating to think that someone has to clean up for them when they're used to doing everything for themselves" *[Admitting Doctor]

Similarly, some participants identified the stoicism associated with people living in the more remote rural areas.

*"I've done some home visits [in very isolated areas] and what I find is that a lot of those people are staunchly independent and going into hospital is like going into another world. They'll fight to not go to hospital and often to their own detriment." *[Community Nursing]

#### Fear

Some of this maintenance of independence played out in not accepting services is also driven by fear of strangers coming into their home. Some participants described this situation as exacerbated now most of the initial contact from various services is made over the phone, particularly in rural areas, whereas in the past the initial contact was made face-to-face and quickly became less threatening as a result.

For others, accessing services is seen to be the first and unwanted step towards life in a nursing home and the associated complete loss of independence and control, as well as the huge change that that would represent, and the frightening association between nursing home care and death.

For some people the idea of ending up in a nursing home did not present a problem, but for others:

*"... it's the bane of their lives, hit me on the head with a brick rather than let me go to a nursing home"*. [Community Nursing]

Some participants had experience of this group of patients agreeing to services and then later declining, and this was a source of frustration. Some felt that patients who were not in any kind of network of services were most at risk of admission. One explanation for this was that:

*"People who are more open to having help seem not to be hospitalised as much because everything is noticed, earlier, faster." *[Pharmacy]

## Discussion

Based on our participants' reports, we suggest there are two overarching categories of barriers which limit or inhibit access to community based services for older patients with chronic disease who have frequent and/or avoidable admissions to hospital. Firstly, 'external' barriers are perceived to exist such as lack of finances, or an actual lack of available services; and secondly, 'internal' barriers were identified such as fear of loss of independence, or a 'stoic' attitude to life, and/or for some, the difficulty of accepting their changed health status.

Whilst we interviewed a broad range of providers (by type, geography and role) it was not an exhaustive list and as our method elicited opinion and often personal reflection and experience, the generalisability of our findings remains open to question. Although participants were able to easily identify and describe external barriers to access, not all described internal barriers. This suggests that this is not a universal phenomenon affecting all patients in this group, affects some patient groups more than others or has variable importance in the perceptions of service providers.

We recognise the need to give a voice to patients directly, and this is an integral aspect of other studies in our project. However, gathering insight from professionals with many years experience of working with countless patients from this group provided a focus for our subsequent studies.

The exploration of barriers affecting access to services for this patient group is not novel. In a recent paper exploring frequent readmissions, Kirby et al state that:

*"...the finding that ACS chronic conditions were associated with frequent readmissions suggests that there are access issues at play." *[2]

Joseph and Cloutier [17] highlight research consistently showing low usage relative to estimated need, suggesting barriers to accessing existing services.

Our findings of external barriers to accessing services reinforce these existing studies. Many participants discussed the impact of rurality on limiting services or making services geographically inaccessible. Rurality has been identified as a factor predicting avoidable ACS hospital admission in a recent systematic review of the literature on determinants of avoidable hospitalisation in chronic disease [5]. Research in Australia also suggests that patients from rural areas are more at risk of an ACS admission than those not in a rural area, implying access to primary care is an issue [7,18]. Slifkin reports that health care seeking behaviour may also differ between rural and urban residents [19]. Kirby et al identified that the location of services, lack of transport to services and waiting times for services were barriers to accessing primary health care in rural areas [2]. One study of older adults in rural West Virginia also reported that some people lack awareness of and accurate information about available services [20].

The significance of socio-economic status (SES) as a factor associated with and predicting frequent and avoidable admission is well rehearsed in previous studies [5,21-23]. Our study has added some detail to the specific mechanisms by which SES (in terms of poor financial status) might influence decisions and behaviour which may lead to frequent and/or avoidable admission. For example, patients deciding not to access services or get a prescription filled.

We have not identified much research however, on the notion of 'internal' barriers to accessing services amongst older people with chronic disease who have frequent and/or avoidable admissions, or indeed much research reporting the patient perspective.

One study offers interesting observations about independence based on interviews with older service-users who:

*"...'cut and pasted' their own solutions to the challenge of maintaining their independence...Tom...arranged his own 'telephone reassurance' with an 83 year old neighbour: she called him in the morning and he called her at night" *[24] p.1043

The researchers report that this maintenance of independence is linked to a strong motivation to stay at home. They also report strong negative responses to the notion of institutionalised care as was identified in our own research.

Two other papers report powerful rural norms and values of independence and self-reliance in relation to service utilisation [19,20]. Whilst this may represent an individual or community's strength, our participants expressed concern that these patients might be more vulnerable to frequent and/or avoidable admission through not accessing services which might reduce the future need for admission.

In a paper exploring uptake of health care amongst the rural elderly of Ontario, Joseph and Cloutier [17] identify a model which provides three elements intrinsic to the decision to utilise services: the degree to which the service is perceived to be important; the perceived quality of the locally available service; and the perceived accessibility, particularly physically, of services. This model whilst potentially useful, omits the significant 'internal' elements of fear, the desire to maintain independence, a stoic attitude, and the difficulty in accepting change and deteriorating health identified by our participants as important influences on the decision to access services. Similarly, in their review of the literature on access, Ansari et al [25] identify five dimensions of access: availability, accessibility (e.g. geographic), accommodation (e.g. design), affordability, and acceptability. Whilst the literature reviewed is from the health care context, and our context is somewhat wider than this, these are useful categories reflecting our own findings of the external barriers to access. However, these categories continue the focus on "supply" and, by implication, on "need" for services but not necessarily the patient perspective on "demand" for services. The possible exception is 'psychosocial factors' included under the *acceptability *dimension (following Anderson [26]), although this refers to ethnic background, class and culture which fails to adequately reflect our interesting findings about internal barriers.

Some of the services, in particular occupational therapy and psychological therapies, which might help such patients with these internal barriers are themselves difficult to access for a number of reasons not least because these services may not be available in rural areas in the way that they can be in urban areas.

It may also be that services themselves exacerbate patient reluctance. One of our participants made the point for example, that the vast majority of services are marketed and approached (by providers and patients) as services designed to prevent further decline rather than actively promote improvement in mental or physical health status. They stated that the system of care is

*"...not set up to enable somebody to improve..." *[Home-based aged care provider]

WHO 2008 World Health Report states that *"...barriers to accessing services are important factors of inequity" *[27] p. xvi. Our participants described a complex set of circumstances underpinning a range of barriers to accessing services for these patients which contribute to inequity and may be contributing to avoidable admission. This reflects the observation of Muenchberger et al that determinants of avoidable hospitalisation are multiple and interacting [5].

**Figure 1:**
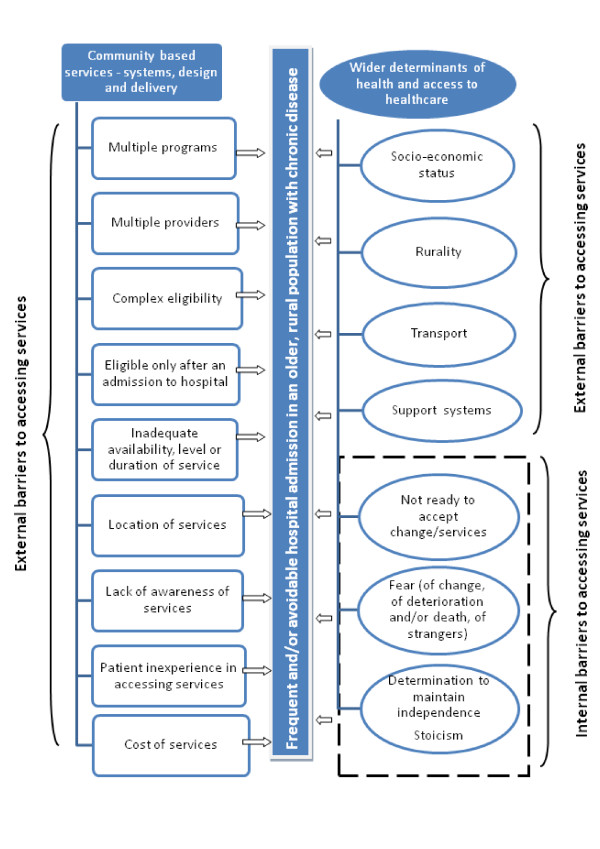
**Barriers to accessing services identified by community based service providers**.

The findings of our study have implications for developing health services for older patients with chronic disease. Many of the barriers to accessing services identified by our participants relate to service systems, design and delivery, and others reflect the wider determinants of health (see Figure 1). Additional resources for community-based services both clinical and non-clinical, would address some of the issues identified, as would (for some patients) some mechanism for navigating the complexity of services. However, the problematic context for such changes includes difficulties with recruitment and retention of staff in rural areas, and the political ramifications of any reallocation of resources.

Without careful exploration of patient, carer and provider perspectives it is difficult to identify the full range of barriers inhibiting access and the interaction between them.

## Conclusions

Access to community based clinical and non-clinical services for rural patients over 65 with ACS chronic conditions is complex. Our findings illustrate some of that complexity. The numerous potential external barriers to accessing services include: the intricacies of provision; patients' lack of awareness of different services and their inexperience in accessing services, patients needing a higher level or longer length of service than they currently have access to, a lack of available services, patient poverty, rurality, and transport. These barriers are potentially further exacerbated by important internal barriers which include fear, the fight against losing independence, a 'stoic' attitude to life, and for some, the confrontational nature of their changed health status.

It may be that simply increasing the amount of services, the "more of the same" approach, implied by much of the literature on ACS and frequent/avoidable admission, whilst addressing some access issues, may well not directly address other external or internal access issues identified here.

We agree with Kirby et al's conclusion that *"...access factors such as, availability, accessibility, accommodation, affordability and acceptability*, *need to be further explored before we have an answer on the impact on frequent readmissions." *[2]

In designing or enhancing services to address frequent and/or avoidable admission (including how those services are delivered), it is key to firstly elicit and understand a range of perspectives on the issue, including those of patients. This is an important component of our continuing work in this area. If the reports of our participants working with this patient group are reflective of patients' thoughts, feelings and behaviours then there exist considerable barriers to accessing services, no matter how comprehensive and apparently accessible they may appear to service providers.

## Competing interests

The authors declare that they have no competing interests.

## Authors' contributions

JL, YG, KH, MP, DE, VS, PC, LB and GM designed the study, JL, YG, GM, KH, LB, DE, MP contributed to the development of the interview topic areas and JL, YG, JS and JP conducted interviews and undertook the analysis. JL and JS produced the first draft of the manuscript and all authors contributed comments and acknowledged authorship. All authors have read and approved the final manuscript.

## Pre-publication history

The pre-publication history for this paper can be accessed here:

http://www.biomedcentral.com/1472-6963/11/265/prepub
